# Application of droplet digital PCR to detect the pathogens of infectious diseases

**DOI:** 10.1042/BSR20181170

**Published:** 2018-11-16

**Authors:** Haiyi Li, Ruolan Bai, Zhenyu Zhao, Lvyan Tao, Mingbiao Ma, Zhenhua Ji, Miaomiao Jian, Zhe Ding, Xiting Dai, Fukai Bao, Aihua Liu

**Affiliations:** 1 Faculty of Basic Medical Sciences, Kunming Medical University, Kunming 650500, China; 2 Faculty of Public Health, Kunming Medical University, Kunming 650500, China; 3 Yunnan Province Key Laboratory for Tropical Infectious Diseases in Universities, Kunming 650500, China; 4 The Institute for Tropical Medicine, Kunming Medical University, Kunming 650500, China; 5 Yunnan Province Integrative Innovation Center for Public Health, Diseases Prevention and Control, Kunming Medical University, Kunming 650500, China; 6 Yunnan Demonstration Base of International Science and Technology Cooperation for Tropical Diseases, Kunming 650500, China

**Keywords:** Droplet digital PCR, diagnosis, infectious disease, Polymerase chain reaction

## Abstract

Polymerase chain reaction (PCR) is a molecular biology technique used to multiply certain deoxyribonucleic acid (DNA) fragments. It is a common and indispensable technique that has been applied in many areas, especially in clinical laboratories. The third generation of polymerase chain reaction, droplet digital polymerase chain reaction (ddPCR), is a biotechnological refinement of conventional polymerase chain reaction methods that can be used to directly quantify and clonally amplify DNA. Droplet digital polymerase chain reaction is now widely used in low-abundance nucleic acid detection and is useful in diagnosis of infectious diseases. Here, we summarized the potential advantages of droplet digital polymerase chain reaction in clinical diagnosis of infectious diseases, including viral diseases, bacterial diseases and parasite infections, concluded that ddPCR provides a more sensitive, accurate, and reproducible detection of low-abundance pathogens and may be a better choice than quantitative polymerase chain reaction for clinical applications in the future.

## Introduction

The infectious diseases are the illness caused by the infection of certain pathogens [1]. Due to pathogens of latency infection, like Ebola virus, malaria, human immunodeficiency virus (HIV) and tuberculosis, their concentrations in plasma are sometimes too low to be determined by traditional methods like ELISA or blood smear. High sensitivity techniques are required for the cheap, fast and accurate detection of pathogens. Quantitative polymerase chain reaction (qPCR) is now applied as a daily used diagnosis tool that helps to check whether a patient has a suspected pathogen infection [3]. Although qPCR has been widely used for the tests of serum, cerebrospinal fluid, and tissue samples; however, its sensitivity, accuracy, and replicability are still not satisfying. What’s more, qPCR shows extremely inaccuracy in the detection of contaminants in very low concentration, because of the instability in low concentration template amplification. qPCR data from different laboratories or clinical tests are not comparable because of the differences in template quality, PCR efficiency, and experimental condition. As the concentration of pathogens in plasma is correlated to the severity of the disease in certain cases, which further reflects the clinical outcome of treatment, accurate absolute quantification of pathogens provides a more powerful clue to better understand the diseases. Hence, making digital droplet polymerase chain reaction (ddPCR) available to clinical studies may give great advantages to correct diagnosis and effective treatment.

## Droplet digital PCR technology

ddPCR is an improved biotechnology of conventional PCR that can clonally amplify and direct quantify DNA or RNA. The main difference between ddPCR and qPCR as the methods used in quantifying DNA is that the former may be more accurate and sensitive.

### Development and principle of ddPCR

In 1992, the concept of ddPCR was raised by Sykes, which quantifies DNA molecules by the combination of Poisson distribution and diluting templates to single molecule level [2]. The principle of ddPCR is to divide a traditional PCR reaction mixture, which is similar to the Taqman® assay, into smaller reaction system either by diluting to microwell plates, capillaries or oil emulsion [3]. Then these small reactions are run individually. After the run, positive reactions are checked and counted among all reactions. Using Poisson’s law of small numbers, the number of templates is positively correlated to the positive wells, so the exact copy number of template can be calculated. The ddPCR can be used to detect low DNA concentration. Other applications like detecting pathogens, gene mutation, gene copy number variation, mRNA expression level, and DNA modifications are reported in recent years. Nowadays, with the requirement for accurate quantification and the development of ddPCR, Bio-Rad®, Life Technologies® and RainDance® are now providing ddPCR machine for laboratories and hospitals [4,5], which provides a better way for nucleic acid detection. The ddPCR analysis is now available to all users and can be adapted to their specific requirements according to instructions and well-organized testing.

### Advantages of ddPCR

**Figure 1:**
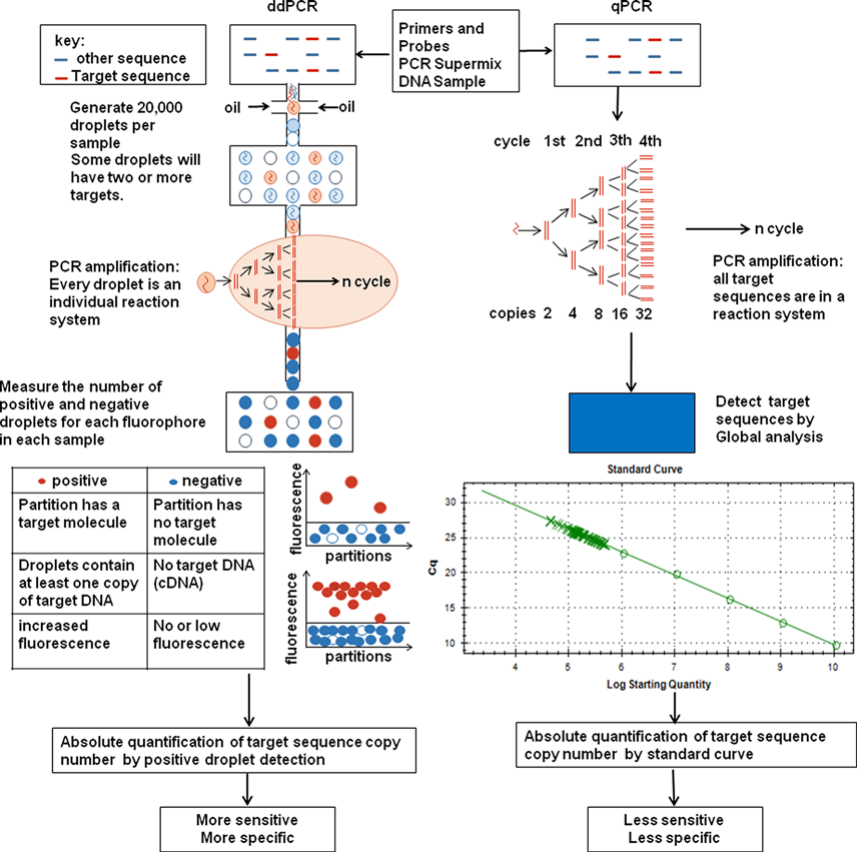
Comparisons of the flowchart and principle of ddPCR and qPCR In droplet digital PCR, the sample is partitioned into 20,000 nanoliter-sized droplets that were individually analyzed. But for qPCR, a single sample offers only a single measurement like traditional PCR. This partitioning in ddPCR process enables the measurement of thousands of independent amplification events within a single sample, so the droplet digital PCR provides an absolute count of target DNA copies per sample without the standard curve when qPCR calculates the target DNA number based on standard curve.

In the request of precise medicine, accurate measurement of pathogen-related nucleic acids is becoming more and more important than ever before. qPCR evaluates DNA amounts by measuring PCR amplified fluorescent signals at certain time point (Cycle threshold, CT), compared to ddPCR which divides templates into individual reaction systems, then transitional PCR are run in individual wells and DNA contains can be quantitated by direct counting PCR positive percentage among all reactions.The traditional qPCR quantifies samples by comparing their CTs to a standard curve generated by well-defined samples, thus qPCR determines sample concentration by an ‘analog’ method. The use of presence and absence of signals to indicate target DNA makes a ‘digital’ direct measurement of samples. The advantage of ddPCR is that it is absolute quantification, does not need a standard curve, and is highly replicable [6,7]. Because of the diversities in sample preparation and PCR condition, even with a standard curve, data diversity in qPCR is higher than ddPCR. The ddPCR counts absolute DNA amounts by direct counting positive wells, which provides better comparable results in different tests. The ddPCR shows higher sensitivity and does not require a pre-enrichment for templates in extremely low concentration [7]. Collectively, ddPCR generally gives higher sensitivity, better accuracy and more stable replications, and recent reports did show the application potential of ddPCR in clinical diagnosis (Figure 1).

## Bringing ddPCR into clinical use for infectious disease diagnosis

Since the first ddPCR machine had been established, scholars tried to bring it into clinical use for years [8,9]. The qPCR is a rapid test method and auxiliary examination in some infectious diseases, but it cannot be the diagnostic standards in some cases because of its low positive and sometimes false positive rate [7]. A study showed that ddPCR was more sensitive and accurate than qPCR; therefore, it has been finely modified to detect low-abundance nucleic acid [10], which might be more suitable for clinical diagnosis. Now, ddPCR has been used to detect low-abundance nucleic acids in many laboratories. According to the above-mentioned original research works, with the development of ddPCR technique, it will be proven to be a powerful tool in detecting the pathogens causing communicable diseases. Here, we show some examples to emphasize the technique advance in ddPCR (Table 1).

**Table 1:** The examples of infectious diseases (pathogens) detected by qPCR or ddPCR

qPCR	ddPCR
1. Hepatitis B (Hepatitis B virus)	1. Hepatitis B (Hepatitis B virus)
2. Acquired immunodeficiency syndrome (human immunodeficiency virus)	2. Acquired immunodeficiency syndrome (human immunodeficiency virus)
3. Tuberculosis (*Mycobacterium tuberculosis*)	3. Bacterial infections (Styphylococcus, Samonella, and Listeria)
4. Human herpes (Equine herpesvirus 1, Equine herpesvirus 4)	4. Tuberculosis (*Mycobacterium tuberculosis*)
5. Syphilis (*Treponema pallidum*)	5. Human herpes (Equine herpesvirus 1, Equine herpesvirus 4)
6. Bacterial enteritis (*Salmonella enterica*)	6. Malaria (malaria parasites)
7. Pointed condyloma (*Human papillomavirus*)	
8. Tuberculosis (*Mycobacterium tuberculosis*)	
9. Malaria (malaria parasites)	

### Viral infectious diseases

A viral infection occurs when an organism is invaded by pathogenic viruses, infectious virus attaches to and enters susceptible cells [11,12]. Viruses can only replicates inside living cells of other organisms, but they are found in almost every ecosystem on Earth and are the most abundant type of biological entity [12,13]. Their variability is high, especially RNA viruses, usually millions of variations. So, in some cases, it is difficult to make effective vaccine targeting each virus, like HIV. The characteristics of the virus mentioned above contribute to its toxicity, universality, and adaptation. Early diagnosis plays an important role in the treatment of viral infection. A great number of scientists use ddPCR to detect kinds of human disease related viruses, such as human herpesvirus, human Cytomegalovirus, human Influenza, and HIV [6,14–17]. Here, we listed some examples to indicate the advantages of ddPCR in the rapid and accurate detection of viral pathogens.

#### Virus hepatitis

Hepatitis B is an infectious disease caused by the hepatitis B virus (HBV), which is transmitted by exposure to infected blood or body fluids [18]. Most of those with the chronic disease have no symptoms; however, some can lead to cirrhosis and hepatocellular carcinoma eventually [19]. Reliable diagnosis of the initial infection can prevent the patient from worsening effectively. Hepatitis B antigens or antibodies are most commonly biomarkers for the diagnosis of HBV infection in modern hospitals. The hepatitis B surface antigen is the first detectable viral antigen during HBV infection, but it may neither present in an early infection nor may present later in the infection as it is being cleared by the host immune system. So a novel, sensitive, and specific platform that can be used to improve HBV detection is in need. By using ddPCR, Huang et al. [20] determine HBV copy number by DNA templates that were prepared from the corresponding formalin-fixed paraffin-embedded tissues. The copy numbers of HBV are positively correlated with Barcelona-Clinic Liver Cancer classification and tumor-nodes-metastasis [20]. A study shows that compared with the conventional quantitative PCR required at least more than 102 copies of cccDNA or HBV DNA, ddPCR required only 0.54 to 0.594 copies of cccDNA for accurate HBV detection, which shows the fact that ddPCR can directly measure nucleic acids at a single-molecule level sensitively and specifically [21]. Obviously ddPCR shows promising application for HBV detection. Interestingly, it had been also used in diagnosing HBV-related tumor as well [20,21].

#### Human immunodeficiency virus

The HIV, which causes acquired immunodeficiency syndrome (AIDS), induces dramatic CD4+ T cells loss during primary infection, which shows no district differences to general influenza. CD8+ T cells are then activated accompanied by the generation of anti-HIV antibodies, like p24 antibody, which is used as a clinical marker for HIV infection [22]. Due to influenza-like symptoms in the HIV primary infection stage and the non-symptom latencystage, it is hard for the early diagnosis of HIV infection until the happening of opportunistic infection in late HIV infection. Serologic methods is useful in primary or latency stage; however, the uses of antibody make it hard to become a daily available test for the high risk population [22]. qPCR based methods are widely used in the detection of HIV, including linear cDNA, 2-LTR circle, and integrated HIV DNA [23]. Since 2012, ddPCR was proved to be useful in the detection of total HIV DNA, 2-LTR circle, and viral RNA in a number of articles [24]. The use of ddPCR in the detection of plasma HIV RNA indicates a better early diagnosis of HIV infection [25,26]. When compared with qPCR, the application of ddPCR in the determination of disease progression and antiviral treatment by total HIV DNA and 2-LTR circle shows higher sensitivity and accuracy [27–29]. Interestingly, ddPCR can also be used to predict the outcome of HIV infection and disease course by measuring the copy number of CCL4L, which encodes the ligand to CCR5 and acts as suppresser of HIV infection [30]. But still some false positive events had been reported unexplainably when detecting HIV viral load [31]. An optimal designed assay, which has suitable controls, can compensate for its disadvantages. Undoubtedly, with a personalized assay, ddPCR will be a powerful and effective platform for diagnosed HIV.

### Bacterial infectious diseases

Bacteria and Protozoa enter our body from respiratory tract, esophagus, genital tract and so on. Bacteria-derived communicable diseases are sometimes hard to be identified, due to the specific biological stages of a specific pathogen, in which the pathogens concentration becomes very low to hide away from being cleaned by immune system. As ddPCR shows good sensitivity to low-abundance DNA, the attempts are made in the detection of pathogens in bacteria-derived communicable diseases.

#### Lyme disease

Lyme disease, transmitted to humans by the bites of *Ixodes ticks*, is a bacterial infectious disease caused by *Borrelia burgdorferi*. The early symptoms may include expanding skin area of redness, rash, fever, headache, and feeling tired. Symptoms may then develop into the loss of ability to move one or both sides of the face, joint pains, heart palpitations, or severe headaches with neck stiffness [32]. The disability rate that mainly caused by misdiagnosis can be up to 60%. Due to these untypical symptoms, it may easily be misdiagnosed as multiple sclerosis, Crohn’s disease, HIV, or other autoimmune and neurodegenerative diseases. For a better treatment of the patients, a better method to make an accurate diagnosis in the early stage is crucial. The most widely used test is serology, which measures levels of specific antibodies in the patient’s blood. But it is only considered as an auxiliary diagnosis of later stages of Lyme disease, because of the low antibody concentration in blood [33]. Now the most widely available and employed serological laboratory tests are ELISA and Western blot. ELISA test is performed first, and if it is positive or equivocal, then the more specific Western blot is run [34]. However, the former test has an overall accuracy at approximately 64%, while the latter is 94% [34,35]. PCR tests for Lyme disease have also been developed to detect the DNA of *Borrelia burgdorferi*. As it often shows false negative for blood and cerebrospinal fluid specimens, PCR is not widely used to diagnose Lyme disease. However, it is still an available test to confirm Lyme arthritis for its higher sensitivity to detect ospA DNA in synovial fluid [36,37]. Even though there is no reported application of ddPCR to the samples of patients with Lyme disease; recently, the potential advantages of ddPCR to detect the pathogens of Lyme disease in ticks were reported [38].

#### Tuberculosis

Tuberculosis (TB), generally affecting the lungs, is a infectious disease caused by the bacterium *Mycobacterium tuberculosis* (MTB). It will not have symptoms in the early infections, in which case it is known as latent tuberculosis. Approximately 10% of latent infections progress to active disease, which, if left untreated, has 50% mortality. Despite the fact that we can prevent TB with bacillus Calmette-Guirin vaccine [39], there are still many people gain TB, especially in developing countries. Multiple sputum cultures for acid-fast bacilli are typically part of the initial evaluation; however, the slow-growing organism always take 2–6 weeks for blood or sputum culture. So quantifying the DNA of *Mycobacterium tuberculosis* with PCR is a robust reproducible method to diagnose tuberculosis. Actually, PCR is becoming a diagnostic tool for pulmonary tuberculosis (PTB) recently. Unlike qPCR, ddPCR can provide a direct count of the bacterial number of the sample, which can improve sensitivity for the detection of tuberculosis precisely [40]. Studies showed that ddPCR can have higher capability to detect a small number of cell-free DNA targets in human plasma [41]. Sufficient specimens of some special patients are hard to be obtained, such as extra pulmonary tuberculosis patients without respiratory lesions, disseminated tuberculosis, infants or children. These cases should obtain samples by invasive procedures such as bronchoscopic examination or surgery in the past because they can only be screened by sputum culture. But now, we can detect MTB-specific DNA fragments in human samples from tuberculosis patients by ddPCR, according to our report [42]. With the development and improvement in the performance of ddPCR, we can diagnose TB more speedily and precisely in the future.

### Parasitic infectious disease

Malaria is a mosquito-borne infectious disease, transmitted to human by an infected female *Anopheles mosquito*. Malaria is usually confirmed by the microscopic examination of blood smears or by antigen-based rapid diagnostic tests (RDT). A parasite level in the blood of greater than 100,000 per microliter in low-intensity transmission areas or 250,000 per microliter in high-intensity transmission areas can be diagnosed as malaria. The accuracy of blood smears ranges from 75 to 90% in optimum conditions to as low as 50% [15]. RDT, by contrast, are often more accurate at predicting the presence of malaria parasites. But they are widely variable in diagnostic accuracy and specificity depending on the manufacturer, and are unable to tell how many parasites are present [16]. In order to increase the analytical accuracy and quantification across a wider range of parasite densities, in the past decade, PCR for malaria have been developed. In spite of the wide application of qPCR, it is difficult to quantify parasite density because of the variety of genome number in different infectious stages and extremely low template concentration in some cases, which makes a difficulty for the comparison among laboratories or hospitals. To overcome such problems, Koepfli et al. first use ddPCR to absolute quantify human malaria parasites. In their study, they show the possibility for potential utilities of ddPCR in clinical diagnosis. They successfully detect *Plasmodium falciparum* and *Plasmodium vivax* at all common parasite densities in humans, and the sensitivity of ddPCR to detect *P. falciparum* is significantly higher than qPCR. Moreover, diversity in ddPCR between technical replicates is 1.6–3.6-fold lower than qPCR, which provides better comparability of results from different groups [43].

## Perspectives

The ddPCR can be used to measure the actual number of molecules as each molecule is in one microdroplet. Compared with qPCR, it provides absolute quantification and is more sensitive and specific for low-abundance DNA (Figure 1). Now, ddPCR had been used in many area, such as to identify a rare allele in a developed heterogeneous tumor, measuring gene copy number variation analysis and analysis of methylation loci [6]. Also, it can be a possible surveillance tool for illnesses such as cancer, and as a vital front end to determining genomic content [9,44]. The advantages of ddPCR lays in many aspects. Technically, ddPCR is more accurate and sensitive, as shown by many studies. More importantly, it gives great benefits to patients in fast and easy diagnosis. On one hand, sampling may be easier in some hard to be accurate diagnosis diseases in the past, like extrapulmonary tuberculosis, because complicated and low template containing samples are now available with ddPCR [42]. On the other hand, PCR-based techniques provide a faster readout than serological or pathogen culturing tests, and ddPCR overcomes the problems in accuracy of qPCR, which makes it more suitable for clinical usage. The ddPCR is now also a potential diagnostic method in infectious disease. However, the reagents and ddPCR machines are still at high price; hence, it needs a period of time to cut down the price and make it a common tool in laboratories. Still, there is no clinical application of ddPCR in the diagnosis of infectious diseases. More clinical samples are needed in testing the performance of ddPCR. And most importantly, the cost of ddPCR machines and reagents are relatively high compared with qPCR, which makes it hard to be spread among general laboratories in facilities or in hospitals (Table 2). In the future, when a ddPCR test is finally come to an affordable price, there will be more applications of ddPCR in hospitals and laboratories, by which a more sensitive, accurate and reliable experimental data or clinical diagnosis result may be produced.

**Table 2:** Advantages and disadvantages of ddPCR

Advantages	Disadvantages
1. Its sensitivity, accuracy and replicability are satisfying	1. Less advantage in an affordable price;c ost of ddPCR machines and reagents are higher
2. Accurate absolute quantification of pathogens	2.Clinical application of ddPCR is still not popular; there are less references available
3. Extremely accurate in very low concentration much less contamination	
4. Sampling may be easier in some hard to be accurate diagnosis diseases	

## Abbreviations

ddPCR: droplet digital polymerase chain reaction
HBV: hepatitis B virus
qPCR: quantitative polymerase chain reaction
RDT: rapid diagnostic tests
